# Knowledge of the nurse in advanced life support and the impact of continuing education in cardiopulmonary arrest in the ICU

**DOI:** 10.1186/cc12934

**Published:** 2013-11-05

**Authors:** Daniella Fernandes Mendonça, Denise de Fátima Gomes Machado, Renata Cristina Barbosa Silva, Camila Medeiros Cruvinel Cunha, Marislei Espíndula Brasileiro

**Affiliations:** 1 Unidade de Terapia Intensiva pelo CEEN, Hospital Santa Genoveva, Bairro Brasil, Uberlâdia, MG, Brazil; 2 CEEN/PUC, Goiânia, GO, Brazil; 3 Saúde Pública e da Família pela UNIASSELVI, UTI Neonatal e Pediátrica, Hospital Santa Genoveva, Bairro Brasil, Brazil; 4 Docente do CEEN/PUC, Goiânia, GO, Brazil

## Background

The knowledge of the nurse in advanced life support and the impact of continuing education in cardiopulmonary arrest in the ICU. Faced with the complications of cardiopulmonary arrest (CPA) in the ICU it is important for nurses to be prepared for emergency actions, mastering the techniques of care and maintaining well-trained staff. The objective was to identify and describe the knowledge of the nurse in advanced life support (ALS) and the impact of continuing education PCR in ICU.

## Materials and methods

Exploratory, descriptive bibliographical integrative analysis of available literature, using the keywords 'PCR', 'ICU', 'Continuing Education', 'Education', 'Nursing', which were published between 2002 and 2012, in both conventional and virtual libraries.

## Results

Twenty-six publications found and gave rise to two categories. First, the identification of clinical signs and CPR maneuver by the nursing staff and the nurse in the PCR are essential for successful resuscitation: the authors agree that the service systematized-based SAV protocol is essential for there to be success in CPR. Recognition theoretical and practical skills of the staff are among the most important determinants of the success rates of RC [1]. Thus, it is necessary that health professionals, especially nursing staff, be aware of the clinical signs of PCR. Furthermore, the residence time of the professional nursing staff in the ICU causes them to gain more experience, making it easier to identify clinical signs and cardiac rhythms [2,3]. Second, the impact of continuing education on quality of nursing care in a PCR: the proper training of the nursing staff, especially those that operate in the ICU, is vital for emergency treatment PCR. Identifying the theoretical and practical knowledge of staff about the PCR and PCR is an important prerequisite for planning a training service [2]. The nurse as team leader and organizer of the ICU is the right professional to establish measures to be taken at the time of the PCR. The nurse has a responsibility to properly distribute the measures to be implemented at the time of service of the PCR, identifying it early and minimizing damage [4].

## Conclusion

Continuing education has significant impact in improving the level of knowledge of nursing professionals, leading to survival of patients in a hospitalized ICU, as it ensures the identification of the signs and symptoms of CRP in patients in the ICU.
